# Supplementary figures and images for: Dense and distributed neuropeptide network in the nerve net of Hydra vulgaris
Source: PLoS Comput Biol. 2026 Mar 20;22(3):e1014037. doi: 10.1371/journal.pcbi.1014037 (PMC13004499; doi:10.1371/journal.pcbi.1014037)

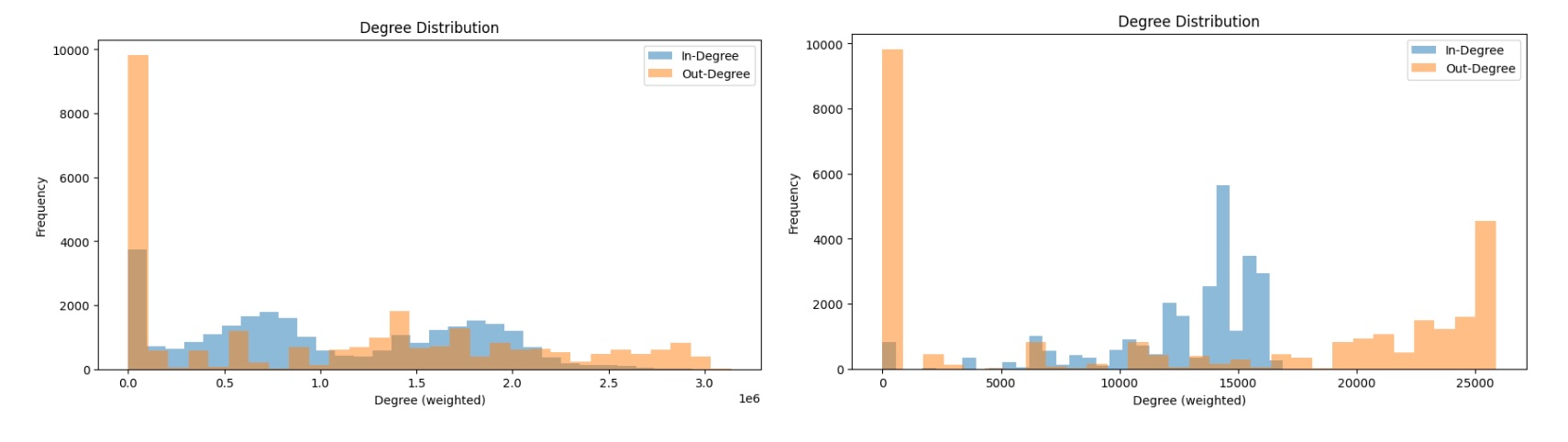

Supplement: S1 Fig — The weighted out-degree distribution is more dispersed and exhibits higher variance, indicating that some neurons receive disproportionately strong inputs compared to others. The weighted in-degree distribution shows a bimodal pattern, suggesting the presence of specialized hub-like neurons with particularly high incoming signals. (Right) Unweighted degree distribution showing the count of connections regardless of strength (i.e., the number of edges), also separated into in-degree (blue) and out-degree (orange). The incoming distribution is more compact indicating a more uniform spread of connectivity when only the presence or absence of connections is considered. (TIFF) [file pcbi.1014037.s001.tiff]

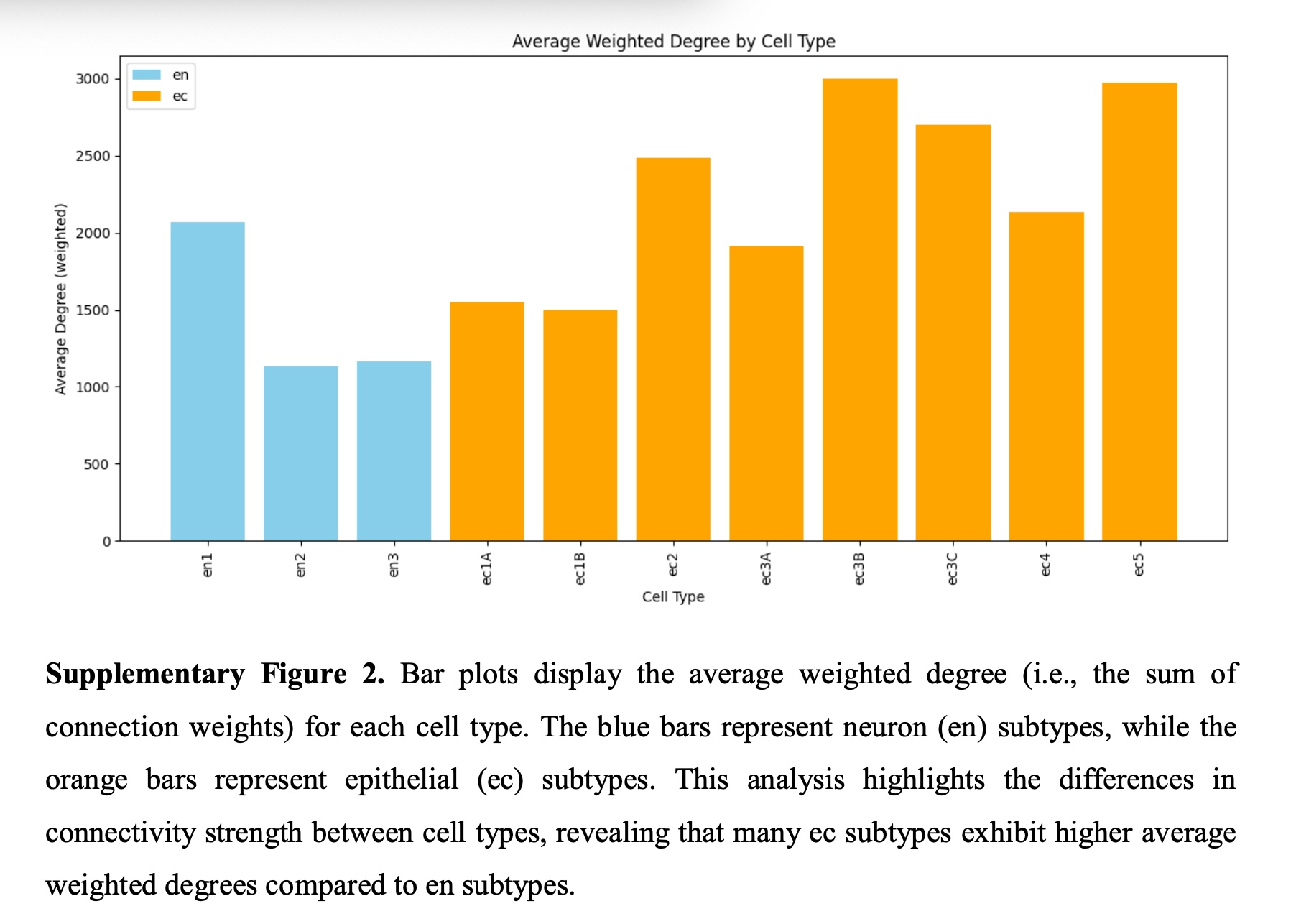

Supplement: S2 Fig — The blue bars represent neuron (en) subtypes, while the orange bars represent epithelial (ec) subtypes. This analysis highlights the differences in connectivity strength between cell types, revealing that many ec subtypes exhibit higher average weighted degrees compared to en subtypes. (TIFF) [file pcbi.1014037.s002.tiff]

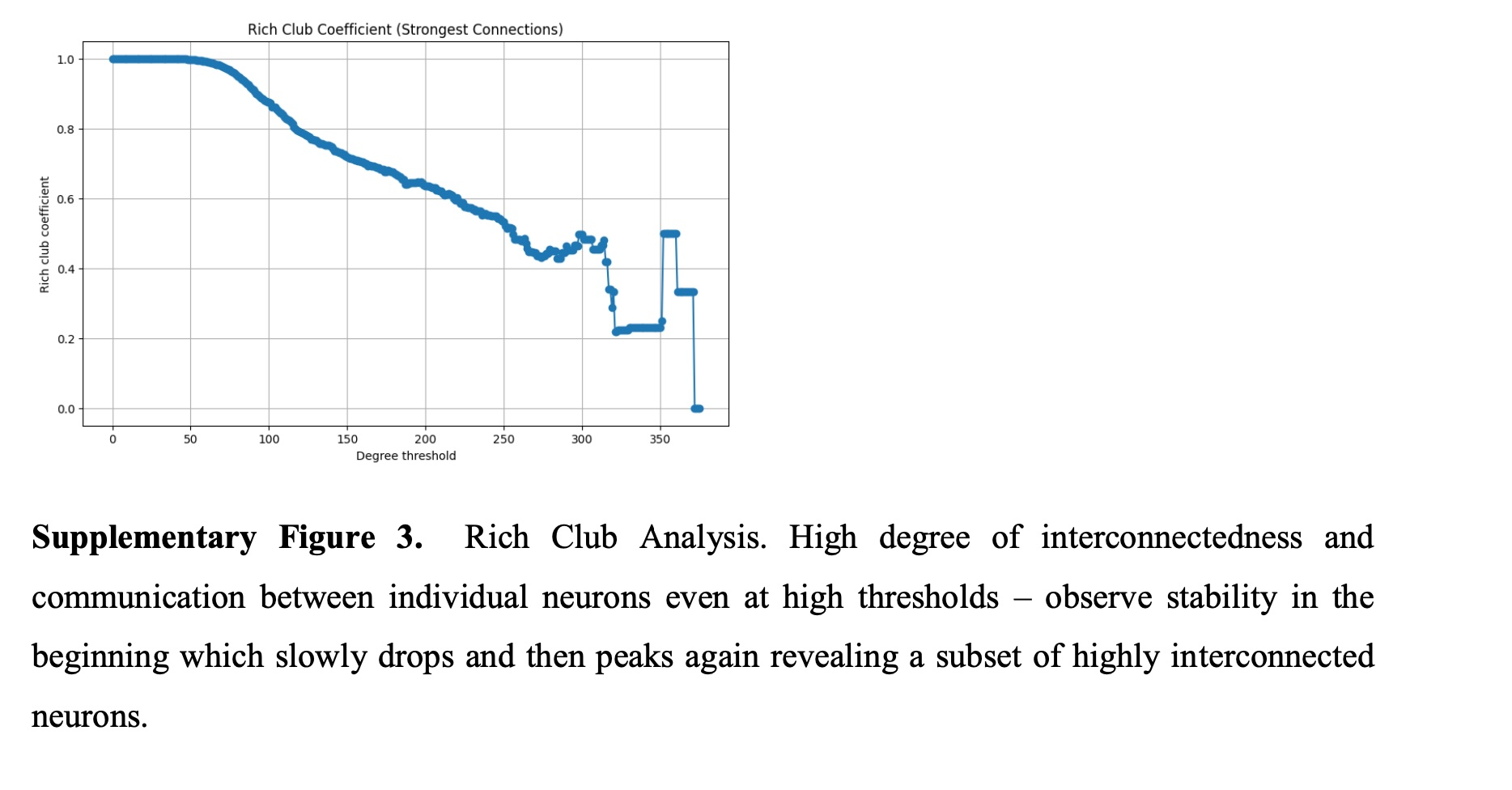

Supplement: S3 Fig — High degree of interconnectedness and communication between individual neurons even at high thresholds – observe stability in the beginning which slowly drops and then peaks again revealing a subset of highly interconnected neurons. (TIFF) [file pcbi.1014037.s003.tiff]

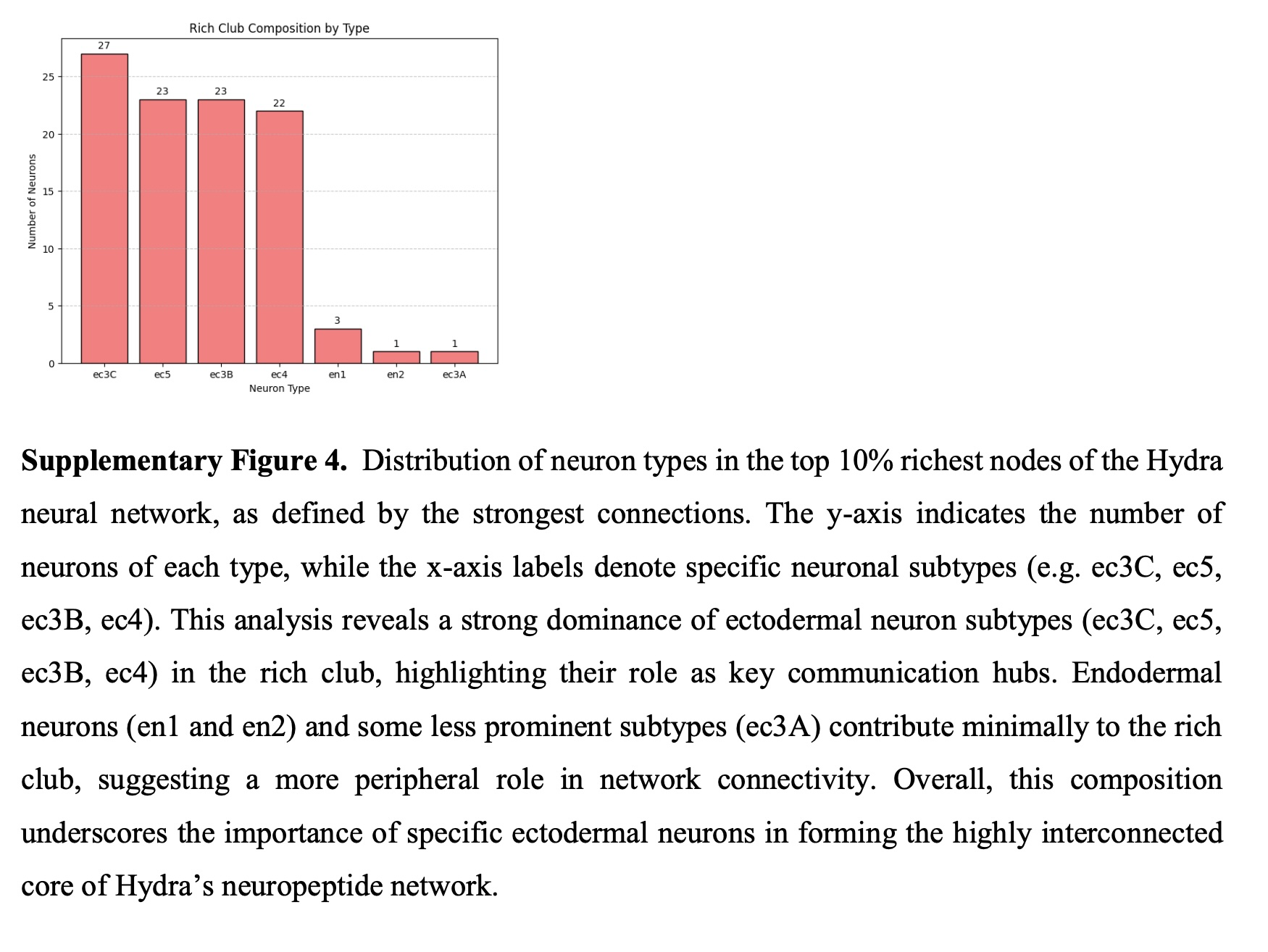

Supplement: S4 Fig — The y-axis indicates the number of neurons of each type, while the x-axis labels denote specific neuronal subtypes (e.g., ec3C, ec5, ec3B, ec4). This analysis reveals a strong dominance of ectodermal neuron subtypes (ec3C, ec5, ec3B, ec4) in the rich club, highlighting their role as key communication hubs. Endodermal neurons (en1 and en2) and some less prominent subtypes (ec3A) contribute minimally to the rich club, suggesting a more peripheral role in network connectivity. Overall, this composition underscores the importance of specific ectodermal neurons in forming the highly interconnected core of Hydra vulgaris neuropeptide network. (TIFF) [file pcbi.1014037.s004.tiff]

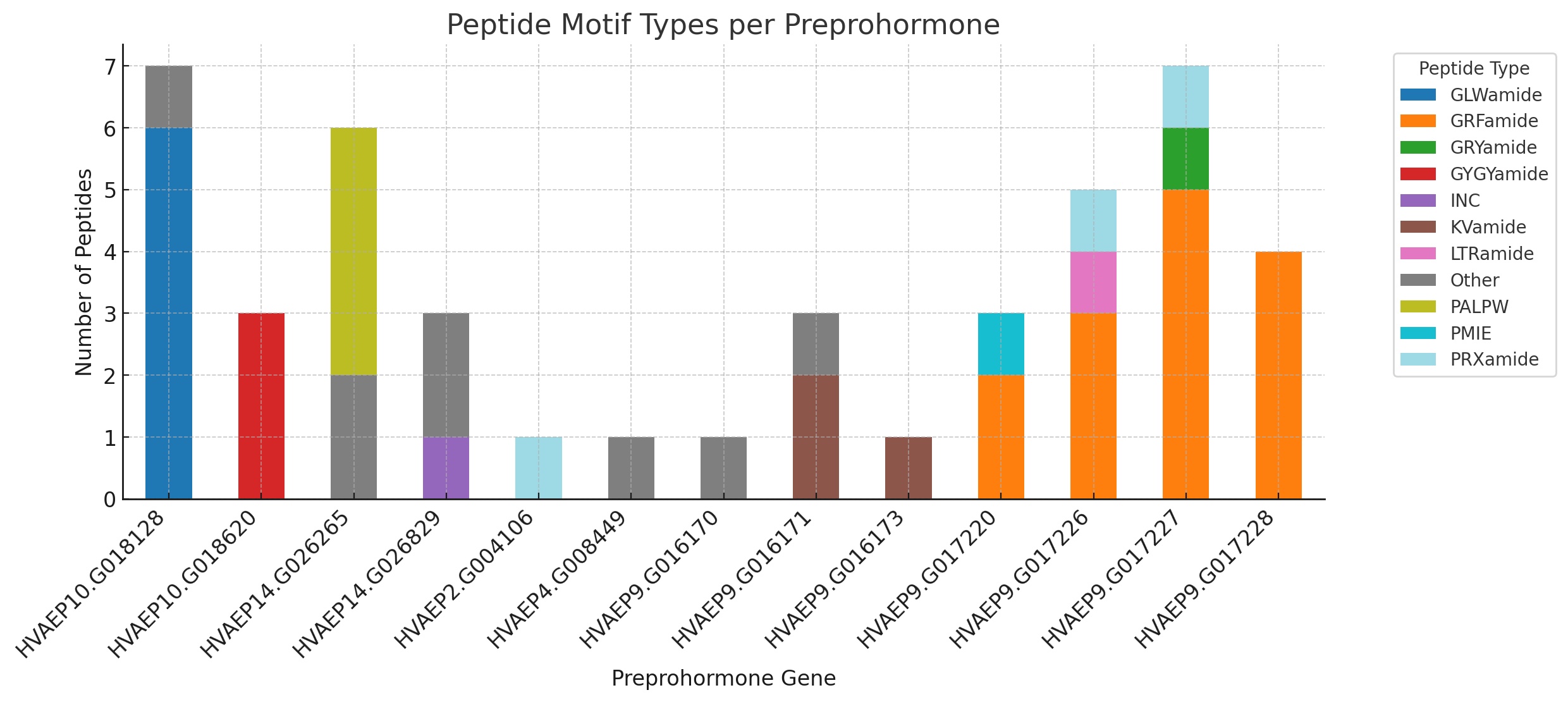

Supplement: S5 Fig — Bar plot showing the number and types of predicted peptide motifs encoded by some of the preprohormone genes. Colors indicate distinct peptide motif families, including GLWamide, GRFamide, GRYamide, GYGamide, INC, KVamide, LTRamide, LPW, PMIE, PRXamide, and peptides assigned to the “Other” category. Each bar represents one preprohormone gene, and stacked segments indicate the number of peptides belonging to each motif class. The plot highlights strong family-specific biases—for example, GLWamide-rich preprohormones (HVAEP10-G018128) and GRFamide-dominated preprohormones (HVAEP9-G017227, HVAEP9-G017228)—illustrating the functional specialization of different peptide-encoding loci. (TIFF) [file pcbi.1014037.s005.tiff]
