# Supplementary material for: Dense and distributed neuropeptide network in the nerve net of Hydra vulgaris
Source: PLoS Comput Biol. 2026 Mar 20;22(3):e1014037. doi: 10.1371/journal.pcbi.1014037 (PMC13004499; doi:10.1371/journal.pcbi.1014037)
Supplement: S2 File — It includes the same information shown in Fig 1, provided here in an extractable format for reuse and further analysis. (DOCX) [file pcbi.1014037.s007.docx]

Neuropeptide Precursor Sequences
>>t1679aep.1_split.1/HVAEP1.T016173.1 (Hym172/176/357690) Hym-176 Family
MSKINKLTMYVFYAFLVLNIYVVLSVNSLPLRDDEDTDEIDGDISELENEYQTNQVYDYNKFKNQADLKVKSRNHYAPFIFPGPKVGRDVNFHSVLSPSDESRKSFNTYYENGYQHDKPAFLFKGYKPGDQTQKNL
> t2059aep.2_split.1/HVAEP9.T017227.1 (RFamide, RFamideII, RFamideV, RFamideVII, RFamideVIII) RFamide Family
DPVEKLKMLSNKKVKLLFALVLIVVEVVKSDDKNFSLEVNKDVKRFIKDILDAKSEEQLMSGRFGKSLPDEEDIDNEVENEYDNEYDDETESQGIINGRYGRQLLRGRFGRQNDNKAASKESQWLGGRFGKEVATQWFNGRFGREIGGRFLPRFGREFNKPHYRGRFGRVAKL
> t3809aep.2_split.1/HVAEP1.T012292.1 (RFamide I , RFamideII, RFamideIII, RFamideV, RFamideVI) RFamide Family
KKKVKMLSNKKVELLFALVLVVVAVVRSEDKNLLLEDNKDVKRIVNDYLETKNGEQLMSGRFGKRETDEGDSDDEDSSEYENEYDDELENQGLANVRYERQLMRGRFGREKNAVSNEDQWLGGRFGREAATQWFNGRFGRDIEGRFLPRFAKESNKPHLRGRFGRAAKL
>t7664aep.3_split.1/HVAEP1.T004115.1 (Hym-355) Hym-355 Family
MMRTAVFGCFILFTIVLALPYRDAFDLFDRFDEYIEKVAKVTADEARLLRDVRNFYKLTKENFVSNADEDDFQDYAPRGGKRENRPRPGK
>t11055aep.1_split.1/HVAEP1.T018128.1 (Hym-53/54/248/249/331/338/370/1071) GLWamide Family
MGMFERKKIVLLVSLICVSQQAANVQDANSKSTSTELKVVKPQKRVTPVKDAEKLSILRTQDNSLDLNTNGEEVWDELTHNIPLEYIEKIYNELNQLAQNENRPKRLWGATAAINTDNLNPEVENELENKKNAPVIEKFERPIGLWHKDVETKNPENRLPLGLWGKDSEPLPIGLWGKDADVNDDLKKEPLPIGLWGKDIDSTQEDNKPNAYKGKLPIGLWGKDNALTNDFGKKNNGKDSGPPPGLWGKDSKPIPGLWGKDNGPMTGLWGKKDVGPPPGLWGKKDQPPIGMWGRAGKKDSNPYPGLWGKKEEEIENVDKEFKEDSLEEYPACLFENPPCEIQEKRYKIEKSGPPPGLWGKRSEKYSMNKPPWRGGMWGRSEILENSVHDSKQTNTIDMEHAEN
>t12588aep.1_split.1/HVAEP1.T016171.1 Hym-176 Family
QVFIVQLKKKKMSKANKLTAFNILLVLNIFVILAVNSLPLRDDEEIDSEIDGDITELENGYQNTQINSYDRHKKQLNPKDKNKKFMIFQGPKVGRDVDFHSVQSPSNKVGKSTRFYYGNDYR
>t12874aep.2_split.1/ HVAEP2.T004115.1 (Hym-355) Hym-355 Family
KMLSLTVATLLLITSIVMAMPNRDATDSNESDILNILDEYIVKVAEMTANEAKILNDVRNYYNDRSSKSLGEFPQSFLPRGGKRDARPRAGK
>t16657aep.3_split.1/HVAEP1.T017226.1 (RFamideII, RFamideIII, RFamideV) RFamide Family
KVSECNHHQVKKKVKMLNHKIETLLVWGLIIVAVVKSEDKNLSAEDRKDVKRIVKDYLNIKNGEQLMSGRFGKRVTDEDIDNEIESEYENEYEDELENFANGREDAAQWFNGRFGREIGGRILPRFATESNKPHLRGRFGRAAKM
>t17992aep.2_split.1/HVAEP1.T016170.1 (Hym-357/690) Hym-357 Family
NEGKMSKVKKLCEFNIILVLYIFLVFSVNALPFKDDEETGIEFDGNISESGNEYQSNQYYDYNKIKNQIYNDYPNIIEKNFKPLKVMKMGRGANDHFDQIGSRKSNDVNLINGNQQDKPAFLFKGYKPGDQTQKKS
>t21435aep.2_split.3/HVAEP1.T008452.1 PNG Family ERKMTRATLAIFFLAILLVIIENNVADRKSHTRHPSIRPSKSVPNGGRPTSLKPSKSETSGRRTPSIKPSKAGGNGKRHSSIKPSKPVPIQSSRNIDSNRDKNKNHKDKKSFGRGHKSVPNG
>t25706aep.1_split.1/HVAEP1.T017220.1 (RFamide I ) RFamide Family
KMATNMALLAFVFFATSIFMLTKADQNEDNQKYDGIARSLKVLLQNYNEKQEEKSDIQNIIEKFSEYQNTGKTIQRKDNVNPMFEKKDAVEQWLGGRFGRVVYDLLLSEVSKDHKRNDETNPMIEKKDADTENRFNREALEQWFSGRFGLTNHKRNDEVNPMIEKKDSEIENRFNREAIEQWLGGRFGRTVYEFLLSETPEKRKK
>t25807aep.3_split.1/HVAEP1.T02115.1 (Framide1 also Hym-65, Framide2 also Hym-1533) FRamide Family
YEFKGFRPLTWKVDFYKVLCFILRNFLVHLETKMYLRLLLVFFVLQISLQESNVRQLDLGQLIEDYLAKENVRREEFLNKINTEILRYIYELENENKGKKRIEASADKNVLEKVLTEVPSIRESVTSKESNVNKMHNSLDSKSSIRSIPTGTLIFRGKKESNSNNENASEQGAPGSLLFRGKKEPNVKENSKNETEASHGERLQQTERNFLVKTKEYIEKLLNSGEEIV
>t33899aep.1_split.1/HVAEP1.T017227.1 (RFamideII, RFamideIII) RFamide Family
VKMLNQRKVEIFFALALIVVALVKSDDNNHLSEGSKNNHLSEDSKNIKRILKDYLNAKNAEQLTRGKLMKRITNKEDNFENDVENEYENKYDDELKNNGHVSKREDATQWFNGRFGREMGERFLPRFGKELNKPHLRGRFGRNIKL
>t6969aep.1_split.1/ HVAEP10.G018620 GGYGYamide Family
RWFMGRTKLFNVNLLAEAAPFKHAKDIIADTKNRDNDDEDESNREENKDLFSNKLSDYGGGYGYGKKEINEDEDQSEGGYGGYGGYGYGKRETERDLGYGGGYGYGKKEINEVADPWRGGR
>t38444aep.1_split.9/HVAEP1.T026265.1 PW Family
KKNSQNMVFSIRLLLLVLILHFQIYKAEEQVSDNLKSDQTTNEIEELFLKNDVSDREKEKTLNKALNDLKTILNDNYDNYNKRNDKINENQKIQKNMLSEEIQKKNENDNLKNGPNAALPWRKDELSMINSLLKRMESSGLLKNNVNNELKDNKKEELNSPALPWRREKLTINSLFNRMESLGLLKRNVNNDLKDPNAALPWRRDEVSLESLLKLMESIGLHKNNKNNDLKGPNAALPWRRDELSNESFLKRLKSSGLFKDNENKELKSPALPWRRDGLSIESLLKRLESFKDNKNNEFNSPALPWRRDEISIKSLLNKMESLGLLENNENDELKSPALPWRRDEISIKSLLNKMESLGLLENNENDELKSPALPWRKEELSIGSLLKRIELLGLLNENKNLHRGSLMLKKDENSFVNFPTKDSAILRDSAIFDNLKDNTMLENEKEKKDNKNSNINDKIAELFKKFKDYFSTENSSETDF
>t21227aep/HVAEP12.T022823 Phoenixin Family
MSLISKLAVYGLVLATGLSLVPIYFVPKAVPEKYRDIQKVSRKDIVQSEVQPGNMKIWSDPFDRKK


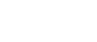


*Legend: red means an identified signal peptide, green a predicted final peptide, yellow identified cleavage sites. The underlined peptides are peptides verified experimentally – both also found in our analysis and not. Further, we have added the names of the determined peptides and their families when possible (families in CYAN).
