# Supplementary material for: Dense and distributed neuropeptide network in the nerve net of Hydra vulgaris
Source: PLoS Comput Biol. 2026 Mar 20;22(3):e1014037. doi: 10.1371/journal.pcbi.1014037 (PMC13004499; doi:10.1371/journal.pcbi.1014037)
Supplement: Attachment — Submitted filename: responseReviewers_december.pdf [file pcbi.1014037.s010.pdf]

Reviewers' comments:

Reviewer's Responses to Questions
Comments to the Authors:
Please note here if the review is uploaded as an attachment.
Reviewer #1: This study constructs a chemical connectome for Hydra vulgaris, offering a well-curated, organism-wide hypothesis space for neuropeptide signaling in a basal metazoan. The resulting resource is both timely and of practical value to the field. Here are my comments:

1. While the network is dense and recurrent, no dynamical modeling is presented. The authors are encouraged to incorporate a minimal dynamical model to demonstrate the potential for multistability within the inferred signaling architecture.
As a response to this review we converted the inferred weight matrix into a simple recurrent dynamical model, where each node updates its activity based on the influence of all other nodes passed through a non-linear activation function (tanh). This allowed us to simulate how the network evolves over time under different initial conditions.
These simulations show that, as predicted, the system reliably settles into stable equilibrium states, and that the network appears to be multistable: depending on the initial conditions, the dynamics converge to one of two distinct final configurations. This demonstrates that the inferred architecture is not only dense and recurrent, but also capable of supporting multiple stable activity patterns.
For computational efficiency, our initial simulations were performed using the simplified version of the connectivity matrix. We can also repeat the analysis using the full matrix if the reviewer considers it necessary. The figures are included in the revised manuscript.

2. There are inconsistencies in the reported numbers of mature peptides and GPCRs. The Abstract and Results sections alternately report 61 and 65 mature peptides (with Fig. 1 caption stating “61”), while the number of GPCRs varies between 65 (Results) and 63 (Methods). These discrepancies should be clarified and corrected across the manuscript.
Thanks for pointing this. With regards to the neuropeptides – the first search resulted in 70 peptide candidates (this was based on finding signal transcripts and looking for cleavage sites)– but of these only 65 fulfilled all criteria – including pattern repetition which resulted in 61 unique peptides – often one peptide was encoded multiple times.
The correct number of GPCRs determined is 65.

3. Please standardize terminology and address editorial issues throughout the manuscript. This includes:

Consistent use of the species name (Hydra vulgaris),

Standardized neuropeptide family nomenclature (e.g., FR, GRF, RFamide),

Correction of typographical inconsistencies (e.g., “GYGY” vs. “GGYG amide”),

Proper use of English possessives (“Hydra’s” instead of “Hydra´s”),

Standardization of month names in references (e.g., “December” instead of “Desember”; “June” instead of “Junie”).
All these typos were identified and corrected across the manuscript.

Reviewer #2: De La Cruz et al. present a computational study of neuropeptide networks in Hydra Vulgaris. The study is interesting and the findings made are potentially very important for comparative neuroscience, but I believe the analyses need to be better documented and justified to be suitable for PLOS Computational Biology. Ideally, in my view, the findings could also be interpreted in more detail with specific cases (e.g. of a putative key network hub) highlighted.

Major points

- the bioinformatic analyses are not sufficiently documented e.g. what versions of tools were used? (sometimes stated, often not). I cannot see code for e.g. the HMM, SignalP, or clans analyses in the Jupyter notebook provided (I haven't assessed it line by line but couldn't find it). Some main headers and/or splitting the notebook into different analyses would be helpful, along with a more detailed 'readme' file.
The GitHub has been expanded with a *readme* file added and the code for the methods where the terminal was used has been included. The code used to run the full analysis has been cleaned and reuploaded with more detail.

- thresholds chosen need to be mentioned and justified (e.g. why 6e-38 for the CLANS clustering?)
We selected an e-value cutoff of 6×10^{-38} because this threshold preserves clear clustering relationships between the *Hydra*´s GPCRs and the experimentally validated *Nematostella vectensis* neuropeptide receptors. Since *Nematostella* is currently the only cnidarian with functional receptor–ligand data, using a threshold that maintains these biologically meaningful relationships can be justified. We now clarify this rationale in the revised manuscript. The other threshold used 10% for the predictions is based on the methods paper that shows that even ones that are not in top 1%, but instead in the top 5- top 10%, are more accurate and better binding in experimental analysis. These clarifications were added into the methods.

- the nature of the underlying data was not clearly explained or linked to (the ID in the relevant database e.g. SRA should be given). For instance the "transcriptome" repeatedly referred to appears to be derived from single cell transcriptomic experiments but the nature of this data and how much of it was used is not clear. The role of the epigenetic map of the Hydra genome cited is also not clear.
We have clarified the origin of the transcriptome data in the methods and the use of the epigenetic maps which provides the source of the newest available and updated single cell atlas of *Hydra* which we used in our interaction analysis.

- it is not clear that the search for homologs based on HMMs is indeed "comprehensive", given the evolutionary distances involved in comparing cnidarians to other groups. A more sensitive search approach such as structure-based approach (ideally with a sensitive method) may well find more relevant GPCRs or neuropeptides. This could be noted as a point for further investigation. Can the implicit claim that all relevant GPCRs have been found been justified? (e.g. were any at the borderline of the thresholds used, which would imply that it is likely that some were missed)
We agree that sensitivity is an important consideration when comparing across large evolutionary distances. However, HMM-based approaches have been shown to perform well in cnidarians. For example, in *Nematostella vectensis*, HMM searches successfully identified 31 neuropeptide GPCRs (e.g., Sachkova et al., 2024). Our pipeline closely follows this established methodology, and we therefore expect HMMs to be sufficiently sensitive for detecting relevant GPCRs in our species. We now describe the steps of our homology search procedure in more detail in the Methods, and we also note that structure-based approaches could be explored in future work.

- the meaning/interpretation of the network properties reported was not sufficiently explained, in my view - how does it relate to other similar networks? Are the results expected? The novelty of the results for Cnidarians is also not quite clear although it seems the claims may be very novel. If so, this can be stated more precisely.
We have added a paragraph to better explain why these results are of wide interest for neuroscience.

- I think more can be said about the putative connection hubs discovered from the network, as this seems a key result. E.g. do neuropeptides/receptors involved in hubs differ from others?
We have studied this more closely and added a supplemental figure showing the distinct neuropeptide groups for every preprohormone. We have also added a more in depth analysis of the significance this might have for the network specificity in the discussion (supplementary figure 5). This addresses directly the specificity of neuropeptide and receptors for distinct functions of the network.

Minor comments

- the work of Cajal & Sherrington is not cited.
We have added those citations.

- Figure 2 - the meaning of the red box is not clear to me, and likewise the titles "GPCR families" and "putative neuropeptide binding GPCR families" are not clear for this figure - which subsets/objects do each refer to?
The red box refers to the GPCRs that fall in the same overall cluster than the experimentally determined neuropeptide binding GPCRs from previous studies. The cluster determination was done by looking at the edges that were above threshold. This was clarified further in the figure legend.

- Figures e.g. Figure 5 need to be at higher resolution.
The resolution has been increased.

- The writing is generally very good but there are a few grammar/spelling issues to fix - e.g. "effectivity of the algorithms", "reciprocals signalling pathways", "show low to none synaptic connectivity".
The grammatic and spelling issues have been removed.

- I don't believe the binding predictions are likely to be accurate, but may be wrong. Is there experimental evidence for a system which is at all similar, using the Alphafold + DeepTMHMM method? Quantifying the expected accuracy here would be useful.
We adapted the AF2–DeepTMHMM peptide–GPCR prediction framework described in Teufel et al. (2023), a peer-reviewed method originally validated on human neuropeptide–receptor interactions. In their benchmarking, the method correctly ranked the true receptor for 11 independent peptides within the top 25 predictions out of more than 1,000 GPCR candidates, demonstrating robust performance in large receptor libraries. In our case, the search space is substantially smaller, and selecting the top 10% of predicted interactions corresponds to an even stricter cutoff than the effective ranking window used in the Teufel et al. validation. Because we do not have experimentally confirmed false interactions for *Hydra*, the accuracy cannot be expressed as a traditional true-positive/false-positive rate; however, our use of a validated prediction system and a conservative top-ranking threshold provides a justified and stringent approach for identifying high-confidence receptor–peptide pairs. We have added these clarifications into the methods to set an expected idea of what experimental accuracy to expect.

Have the authors made all data and (if applicable) computational code underlying the findings in their manuscript fully available?
The PLOS Data policy requires authors to make all data and code underlying the findings described in their manuscript fully available without restriction, with rare exception (please refer to the Data Availability Statement in the manuscript PDF file). The data and code should be provided as part of the manuscript or its supporting information, or deposited to a public repository. For example, in addition to summary statistics, the data points behind means, medians and variance measures should be available. If there are restrictions on publicly sharing data or code —e.g. participant privacy or use of data from a third party—those must be specified.
Reviewer #1: None
Reviewer #2: No: The code provided is not clearly organized and does not appear to all be available
We have reviewed the GitHub and organized it to add the code for the pipelines using HMMER and BLAST as well as making the code more easily understandable.

In particular, we kindly ask you to consider addressing the potential role of epigenetic factors in gene expression, as well as how the proposed network topology might adapt to varying environmental conditions.

This comment was addressed in the limitations and through an extended network analysis – further concluded in the discussion.
